# Evaluation of sequential serum amyloid A measurements for the prediction of sepsis and survival in critically ill neonatal foals

**DOI:** 10.1093/jvimsj/aalag155

**Published:** 2026-08-06

**Authors:** Eva de Bruijn, Mathilde Laetitia Pas, Donatienne Castelain, Alexander Dufourni, Ellen Paulussen, Bart Pardon

**Affiliations:** Department of Internal Medicine, Reproduction and Population Medicine, Ghent University, 9820 Merelbeke, Belgium; Department of Internal Medicine, Reproduction and Population Medicine, Ghent University, 9820 Merelbeke, Belgium; Department of Internal Medicine, Reproduction and Population Medicine, Ghent University, 9820 Merelbeke, Belgium; Department of Internal Medicine, Reproduction and Population Medicine, Ghent University, 9820 Merelbeke, Belgium; Department of Internal Medicine, Reproduction and Population Medicine, Ghent University, 9820 Merelbeke, Belgium; Department of Internal Medicine, Reproduction and Population Medicine, Ghent University, 9820 Merelbeke, Belgium

**Keywords:** biomarker, neonatal septicemia, serial SAA determination, survival analysis

## Abstract

**Background:**

Single measurements of serum amyloid A (SAA) concentration at hospital admission has limited sensitivity for predicting sepsis and death.

**Hypothesis/Objectives:**

To determine whether differences in SAA during the first 2 days of hospitalization could predict sepsis and death in critically ill foals.

**Animals:**

One hundred twenty-seven critically ill neonatal foals, <14 days of age.

**Methods:**

Prospective cohort study. SAA was measured at hospital admission and on day 2 of hospitalization using a validated point-of-care test. Logistic regression was conducted to evaluate the predictive value of individual SAA measurements for diagnosing sepsis and assess whether change in SAA during 48 h could predict sepsis or death. Cox survival analysis assessed the association between SAA concentration and death.

**Results:**

Serum amyloid A (SAA) concentrations were measured on day 0 (*n* = 127) and day 2 (*n* = 97) of hospitalization. On admission and day 2, SAA was not significantly associated with death (*P* = .20 and *P* = .08), but was significantly associated with neutropenia (OR 1.001; 95% CI, 1.001-1.1002; *P* ≤ .001), blood culture positivity (OR 1.001; 95% CI, 1.0-1.1001; *P* = .002), or both (OR 1.002; 95% CI, 1.001-1.1003; *P* ≤ .001), with optimal cut-offs of 419 μg/mL on day 0, with moderate sensitivity and specificity (80% and 81%, respectively). Changes between day 0 and day 2 were not predictive for death (*P* = .49), neutropenia (*P* = .36), blood culture positivity (*P* = .41) or both (*P* = .17).

**Conclusions and clinical importance:**

In this cohort, SAA only had moderate ability to rule in or rule out sepsis or predict death. Repeated measurements after 48 h did not improve accuracy, suggesting that SAA should not be used as a standalone test.

## Introduction

Sepsis is a life-threatening condition in foals.^1^ Clinical signs are often vague with septic shock, multiple organ failure and death possibly occurring rapidly.^2–4^ Treatment is intensive, expensive, and often associated with a poor outcome. Medical care involves the use of antimicrobials.^2–6^ Rapid and appropriate treatment affects outcome,^7^ but definitive diagnosis of sepsis remains difficult. Blood culture is an important component of the standard diagnostic approach, but has low sensitivity.^7^ The delay between sampling and the availability of antimicrobial susceptibility test results often takes 48-72 h.

Research in human medicine has focused on identifying reliable biomarkers for predicting sepsis and death.^8–10^ Serial assessment of biomarkers is useful to assess the severity of disease, predict death, and to assess therapeutic response.^8–13^ In veterinary medicine, several biomarkers have been investigated for their potential to detect sepsis and to predict death in foals, including fibrinogen, serum amyloid A (SAA), haptoglobin, and procalcitonin.^4^^,^^14–20^ SAA is an acute-phase protein typically found at low concentrations in healthy individuals, but its concentration increases markedly within 6-12 h after an inflammatory insult, peaking around 48 h.^4^^,^^10^^,^^17^^,^^18^^,^^21–24^ An increase in SAA concentration might suggest the presence of a systemic infection, warrant further investigation, and aid clinicians in deciding when to initiate treatment.^23^ SAA concentration decreases rapidly once inflammation resolves, making it valuable for monitoring therapeutic response.^17^ A limited number of studies have evaluated the diagnostic accuracy of SAA in predicting sepsis in foals and reported low sensitivities and moderate to high specificities for predicting both sepsis (30%-65% and 68%-90%) and death (20%-22% and 90%-97%), respectively.^17^^,^^18^^,^^25^

Recently, a working group proposed a new definition for foal sepsis as a life-threatening condition involving proven or suspected infection with systemic manifestations of infection.^26^ Further working groups were set in place to develop scoring systems to predict sepsis and risk of death, and to further determine manifestations of infection.^26^ This working group stated that SAA had limited value in the diagnosis of sepsis in foals.^26^ Repeated SAA measurements in critically ill neonatal foals might offer added diagnostic or prognostic value to predict sepsis and survival compared to single measurement upon arrival.^27^

This study aimed to investigate the diagnostic and predictive value of a single SAA measurement versus changes in SAA concentration within the initial 48 h of hospitalization, with a point-of-care (POC) test, for the diagnosis of sepsis and death in critically ill foals, younger than 14 days of age. A second objective was is to externally validate previously determined cut-off values for SAA concentration to predict sepsis in foals.

## Materials and methods

### Study design, sample size, and cohort

A prospective cohort study was performed in foals younger than 14 days referred to the Large Animal Internal Medicine Department of Ghent University between January 2023 and August 2024. The study was approved by the Ethical Committee of the Faculty of Veterinary Medicine and Bioscience Engineering, Ghent University (EC2021/023). Sample size was calculated a priori based on sepsis prevalence of 40%; with a power of 80% and a 95% CI to detect a 35% difference in sepsis risk between 2 categories of a factor, 59 animals were needed per test group. When considering death and sepsis risk, sample size was increased to 127 animals.

A convenience sample of critically ill neonatal foals was included. Critical illness was defined as the presence of neurological dysfunction (lethargy or coma) with or without decreased or absent suckle reflex and need for intravenous fluid support. Foals were defined as septic when blood culture vials tested positive. Two composite definitions were constructed: blood culture positivity, neutropenia, or both, and another including foals blood culture positivity, or SIRS, or both.^26^

Data were collected by 9 experienced equine clinicians, using a standardized evaluation form. A total of 30 mL of blood was aseptically collected for blood culture. SAA concentration was determined on EDTA-anticoagulated whole blood and measured by using a POC test (Stablelab EQ-1 handheld reader, Zoetis B.V., Parsippany, New Jersey, United States) within 30 min after blood collection. On day 2 of hospitalization whole blood was collected in an EDTA-tube to determine SAA concentration. Clinical outcome was defined as survival to discharge or mortality, Including natural death and euthanasia. No distinction was made between foals that were euthanized for medical, prognostic, or financial reasons.

### Statistical analysis

Data analysis was performed in SPSS (IBM SPSS Statistics for Windows, Version 29.0. Armonk, NY: IBM Corp). SAA values were tested for normality. The association of SAA concentration and sepsis was evaluated using univariable and multivariable logistic regression. Receiver operating characteristic curve analysis and the Youden index were used to determine optimal cut-off values for SAA concentration, when significant.

The association of SAA with death was analyzed by univariable Cox proportional hazard (survival) modeling, including sensitivity analysis. The overall significance level was set at *P* < .05. Model assumptions and statistical procedures are described in detail in the Appendix S2.

## Results

### Study sample, sepsis, and risk of death

A total of 127 critically ill foals were included in this study. Data collected on day 2 was only available for 97 foals due to death before to second sampling time point of 25 (83%) foals or missing data for 5 (15%) foals. The median age of all foals at admission was 1 day [interquartile range (IQR), 0; 3], with a maximum of 13 days. Septic foals had a median age of 2 days [IQR, 1; 3] and hospitalized foals had a median age of 1 day [IQR, 0; 3]. In the present cohort, 99 (78%) foals were determined as newborn (< 3 days of age) and 28 (22%) foals were determined as neonatal (4 days to 2 weeks of age).^26^ The study sample included 67 colts and 60 fillies. Duration of hospitalization ranged from 1 to 39 days, with a median of 6 [2.7; 11.0] days. Based on the working group’s consensus definitions, foals were divided into septic (based on different definitions) and hospitalized groups.^26^ Antimicrobial history was known for 123 foals, with 35 (28.5%) foals treated with antimicrobials before blood cultures were sampled, whereas 88 (71.5%) did not receive antimicrobial treatment.

Overall, 83 foals (65%) survived to hospital discharge, whereas 44 foals (35%) died. Of the 44 foals that died, 15 foals (34%) died naturally and 29 (66%) were euthanized.

**Figure 1:**
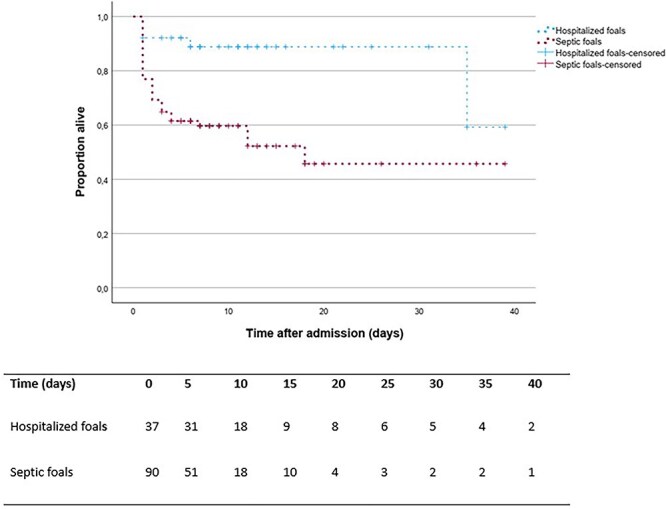
Kaplan–Meier cumulative mortality curves for hospitalized (*n* = 37) and septic foals (*n* = 90) (foals with neutropenia, blood culture positive or both over 40 days). The *Y*-axis represents cumulative mortality probability, and the *X*-axis represents days of follow-up. Censored points represent foals that were discharged alive from the hospital and thus were no longer followed for mortality within the study period. Numbers at risk are shown below *X*-axis. Most deaths in septic foals occurred within the first week, while survival remained relatively stable in hospitalized foals.

Under all 4 definitions the presence of sepsis at hospital admission was associated with a higher risk of death: blood culture positivity (HR: 2.0; 95% CI, 1.1-3.6; *P* = .03), neutropenia (HR: 2.5; 95% CI, 1.2-5.3; *P* = .01), and the combination of neutropenia, blood culture positivity, or both (HR: 3.7; 95% CI, 1.5-10.1; *P* = .004) and SIRS (HR: 6.8; 95% CI, 2.1-22.2; *P* = .001). For SIRS combined with blood culture positivity, analysis revealed a HR: 1.9; 95% CI, 1.0-3.8; *P* = .05. A Kaplan–Meier survival analysis was performed to assess overall survival in foals with sepsis, defined as the presence of neutropenia, positive blood culture, or both. Foals with sepsis demonstrated a progressive decline in survival over time (Figure 1). The cumulative death increased steadily, with a notable separation of the survival curves between septic and hospitalized foals. Most deaths of septic foals occurred within the first week, while survival remained relatively stable in hospitalized foals.

### SAA measurements

Serum amyloid A concentrations on the day of hospital admission (d0) were available for 127 foals, and after 48 hours of hospitalization (d2) for 97 foals. Data are shown in Table 1. Serum amyloid A concentrations on d0 and d2 were not significantly associated with death (*P* = .20 and *P* = .08, respectively). When performing sensitivity analysis, only including cases of natural death, SAA revealed a weak association with death on day of admission (OR 1.001; 95% CI, 1-1.1001; *P* = .05). Multivariable logistic regression identified prematurity as a possible confounder when assessing for death. A sensitivity analysis with exclusion of premature and euthanized foals was not possible due to small sample size. Additionally, the dynamic change in SAA between d0 and d2 (available for 97 foals) was not significantly associated with outcome (*P* = .49). The median change was 57 μg/mL [IQR, −219; 628] for survival and 310 μg/mL [IQR, −136; 820] for foals that died.

**Table 1:** SAA concentration (in μg/mL) of septic vs. hospitalized foals based on different definitions and for survival and non-survival.

	**D0**		**D2**	
	**Number of foals (*n*)**	**Median [IQR]**	**Number of foals (*n*)**	**Median [IQR]**
**Blood culture positive**	*58*	*939* [*548; 1564*]	*38*	*1104* [184*; 1720*]
**Blood culture negative**	*69*	*399* [47*; 1033*]	*61*	*659* [*246; 1720*]
**Neutropenia positive**	*80*	*1016* [540*; 1779*]	*57*	*1181* [*534; 2108*]
**Neutropenia negative**	*47*	*177* [25;748]	*42*	*383* [*103; 814*]
**Neutropenia, positive blood culture or both**	*90*	*1022* [535*; 1839*]	*64*	*1130* [383*; 1997*]
**Neutropenia negative and negative blood culture**	*37*	*74* [13*; 352*]	*35*	*443* [153*; 807*]
**SIRS positive**	*71*	*836* [*257; 1703*]	*47*	*975* [*363; 2028*]
**SIRS negative**	*36*	*638* [25*; 1046*]	*35*	*576* [*160; 1448*]
**SIRS, positive blood culture or both**	*85*	*834* [219*; 1626*]	*61*	*892* [211*; 1836*]
**SIRS negative and negative blood culture**	*21*	*550* [35*; 1030*]	*21*	*658* [354*; 1875*]
**Survival**	*83*	*741* [*93; 1096*]	*83*	*737* [189*; 1512*]
**Death**	*44*	*849* [91*; 1929*]	*14*	*1205* [*657; 2028*]

Abbreviations: d = day; IQR = interquartile range.

Serum amyloid A concentration on d0 was significantly associated with blood culture positivity, neutropenia, and neutropenia with or without blood culture positivity (*P* = .002, *P* ≤ .001, and *P* ≤ .001), the presence of SIRS as a sole variable or combined with blood culture was not significantly associated with SAA (*P* = .11 and *P* = .06) (Table 2). Receiver operator curve analysis was performed for all significant variables and yielded an optimal cut-off value of 419 μg/mL for blood culture positivity (Area Under the Curve (AUC) = 0.68; SE = 0.05; 95 CI, 0.56-0.77) and neutropenia combined with blood culture positivity (AUC = 0.82; SE = 0.04; 95% CI, 0.74-0.90) (Table 3). Neutropenia yielded a lower cut-off value of 379 μg/mL (AUC = 0.74; SE = 0.05; 95% CI, 0.65-.83), predicting neutropenia with a Se 82.5% (95% CI; 74.2-90.8), a Sp of 68.1% (95% CI; 54.8-81.4), and an LR+ and LR- of 2.59 and 0.26. In addition Brier scores were calculated (Table 2).

**Table 2:** Results of logistic regression analyses and brier scores evaluating the association between the biomarker concentration at admission (d0) and day 2 (d2) with different sepsis definitions.

	**D0**					**D2**				
**Variable**	**Number of foals (*n*)**	**OR**	**95% CI**	***P*-value**	**Brier (CI)**	**Number of foals (*n*)**	**OR**	**95% CI**	***P*-value**	**Brier**
**Blood culture positivity**	127	1.001	1.0-1.001	**.002**	0.23 (0.2-0.25)	99	1.001	1.0-1.001	.37	0.24 (0.21-0.26)
**Neutropenia positivity**	127	1.0	1.001-1.002	**<.001**	0.15 (0.12-0.18)	99	1.0	1.001-1.002	**<.001**	0.20 (0.17-0.24)
**Neutropenia, blood culture positive, or both**	127	1.002	1.001-1.003	**<.001**	0.15 (0.12-0.18)	99	1.001	1.0-1.003	**.001**	0.20 (0.17-0.24)
**SIRS positivity**	109	1.0	1.0-1.001	.11	0.22 (0.19-0.25)	82	1.0	1.0-1.001	.21	0.24 (0.22-0.26)
**SIRS, blood culture positivity, or both**	109	1.0	1.0-1.001	.06	0.23 (0.21-0.25)	89	1.0	1.0-1.001	.81	0.25 (0.24-0.26)

Abbreviations: d = day; OR = odds ratio.

**Table 3:** Diagnostic performance of determined cut-off value (419 μg/mL) calculated on the present cohort for different sepsis definitions in the current cohort.

**Diagnostic cut-off value**	**419 μg/mL**		
	**Blood culture positivity**	**Neutropenia**	**Neutropenia, blood culture positivity, or both**
**OR; CI**	4.9; 2.2-11.1	8.5; 3.8-19.4	17.1; 6.5-45.2
**Se (%) (95% CI)**	81.0 (70.9-91.1)	81.0 (72.-89.7)	80 (71.7-88.3)
**Sp (%) (95% CI)**	53.6 (41.9-65.4)	66.7 (53.3-80.0)	81.1 (68.5-93.7)
**Accuracy (%) (95% CI)**	66.1 (57.9-74.4)	75.6 (68.1-83.1)	80.3 (73.4-87.2)
**LR+**	1.74	2.43	4.23
**LR−**	0.35	0.29	0.25

Abbreviations: LR+ = positive likelihood ratio; LR− = negative likelihood ratio; OR = odds ratio; Se = sensitivity; Sp = specificity.

Possible confounding factors were assessed with multivariable logistic regression for the different sepsis definitions, including: age (in days), prematurity, prior antimicrobial treatment, and presence of comorbidity. No significant effects of these variables on the different sepsis outcomes were found (age (*P* = .26), prematurity (*P* = .67), prior antimicrobial treatment (*P* = .37), and comorbidities (*P* = .33). In the multivariable model for sepsis defined by neutropenia prior antimicrobial treatment revealed an association with SAA (*P* = .04), but the interaction was not significant when assessed with logistic regression (*P* = .42). For both neutropenia and the combination of neutropenia, blood culture positivity, or both, prior antimicrobial treatment revealed possible confounding (*P* = .04), but the interaction was not significant when assessed with logistic regression (*P* = .43).

SAA concentration on d2 was not associated with blood culture positivity and SIRS positivity (*P* = .08 and *P =* .21). A significant association was present between SAA concentration on d2 with presence of neutropenia and the combination of neutropenia, blood culture positivity, or both (*P* ≤ .001 and *P* = .001, respectively). Receiver operator curve analysis yielded an optimal cut-off value of 845 μg/mL for neutropenia (AUC = 0.75; SE = 0.05; 95% CI, 0.65-0.85) and 933 μg/mL for the combination of neutropenia, blood culture positivity, or both (AUC = 0.70; SE = 0.05; 95% CI, 0.56-0.81). Data shown in Table 3. The difference in SAA concentration between d0 and d2 (available for 97 foals) showed no significant association with blood culture positivity (*P* = .41), neutropenia (*P* = .36), or both and (*P* = .17), SIRS (*P* = .82) or both SIRS and blood culture positivity (*P* = .95). In the present cohort an external validation of reported SAA cut-off values was done (Table 4).^17^^,^^18^ A substantially lower sensitivity and specificity were found for the 1050 and 100 μg/mL, respectively (Table 5).

**Table 4:** Diagnostic performance of day 2 (d2) SAA cut-off values determined to be significant, evaluated according to different sepsis definitions in the study cohort.

**Diagnostic cut-off value**	**845 μg/mL**		**933 μg/mL**	
	**Neutropenia**	**Neutropenia, blood culture positivity, or both**	**Neutropenia**	**Neutropenia, blood culture positivity, or both**
**OR; CI**	7.9; 3.1-20.2	7.5; 2.7-20.7	6.8; 2.6-17.2	8.7; 3.0-25.6
**Se (%) (CI)**	64.9 (52.5-77.3)	60.9 (49.0-72.9)	61.4 (48.8-74.0)	59.4 (74.3-71.4)
**Sp (%) (CI)**	81.0 (69.1-92.8)	82.9 (70.4-95.3)	81.0 (69.1-92.8)	85.7 (74.1-97.3)
**Accuracy (%) (CI)**	71.7 (62.8-80.6)	68.7 (59.6-77.8)	69.7 (60.6-78.7)	68.7 (59.6-77.8)
**LR+**	3.42	3.56	3.23	4.15
**LR−**	0.43	0.47	0.48	0.47

Abbreviations: LR+ = positive likelihood ratio; LR− = negative likelihood ratio; OR = odds ratio; Se = sensitivity, Sp = specificity.

**Table 5:** Diagnostic performance of published cut-off values of serum amyloid a for the prediction of sepsis based on different definitions in the current cohort.^17^^,^^18^

**Diagnostic cut-off value**	**100 μg/mL** ^17^			**1050 μg/mL** ^18^		
	**Blood culture positivity**	**Neutropenia**	**Neutropenia, blood culture positivity, or both**	**Blood culture positivity**	**Neutropenia**	**Neutropenia, blood culture positivity, or both**
**OR; CI**	2.7; 1.1-6.5	5.1; 2.2-12.0	7.6; 3.1-18.6	3.4; 1.5-7.3	4.5; 1.9-11.7	7.5; 2.7-20.7
**Se (%) (CI)**	84.5 (75.2-93.8)	86.3 (78.7-93.8)	86.7 (79.6-93.7)	48.3 (35.4-61.1)	45.0 (34.1-55.9)	45.6 (35.3-55.8)
**Sp (%) (CI)**	33.3 (22.2-44.5)	44.7 (30.5-58.9)	54.1 (38.0-70.1)	78.3 (68.5-88.0)	85.1 (74.9-95.3)	94.6 (87.3-100)
**Accuracy (%) (CI)**	56.7 (48.1-65.3)	70.9 (63.0-78.8)	77.2 (69.9-84.5)	64.6 (56.2-72.9)	59.8 (51.3-68.4)	59.8 (51.3-68.4)
**LR+**	1.27	1.56	1.89	2.23	3.02	8.4
**LR−**	0.47	0.31	0.25	0.66	0.65	0.57

Abbreviations: LR+ = positive likelihood ratio; LR− = negative likelihood ratio; OR = odds ratio; Se = sensitivity, Sp = specificity.

## Discussion

This study evaluated the diagnostic and prognostic value of SAA measurements in critically ill neonatal foals using a POC assay, with emphasis on repeated measurement after 48 h. With a POC test, using EDTA-coagulated whole blood, this study also aimed to evaluate a technique considered practical for both rapid clinical decision-making in a hospital environment and field application by veterinarians.

Our findings confirm the association between increased SAA concentration at admission and the presence of sepsis.^17^^,^^18^ In the present cohort, the optimal cut-off value yielding the highest combined sensitivity and specificity, was 419 μg/mL for both blood culture positivity and combination of blood culture positivity and neutropenia, which lies between the previously reported thresholds of 100 and 1050 μg/mL.^17^^,^^18^ At the cut-off of the present study, sensitivity was 81.5%, which is markedly higher than the previously reported values of 52.9% (at 100 μg/mL) and 30.2% (at 1050 μg/mL). In contrast, specificity at 419 μg/mL was lower (53.6%-80%) compared to previously reported 97.5% (at 100 μg/mL) and 90.7% (at 1050 μg/mL). When the previously published cut-offs were applied to the current dataset, sensitivity increased to 84.5% and 48.3%, while specificity decreased to 33.3% and 78.0% for 100 and 1050 μg/mL, respectively.^17^^,^^18^ These discrepancies are likely attributable to differences in cohort, sepsis definitions, and analytical methods used to quantify SAA. Given that previous studies used different assays (eg, ELISA, latex agglutination, lateral flow immunoassays), direct comparison of Se and Sp between studies is challenging.^17^^,^^18^^,^^25^ A cut-off value of 100 μg/mL was evaluated using lateral flow immunoassay, whereas the study of Hoebert et al. establishes a cut-off value of 1050 μg/mL using different techniques and laboratories, which were not specified in the article.^17^^,^^18^ The lack of external validation of reported cut-off values further limits generalizability. Consequently, the diagnostic performance of any SAA threshold should be interpreted in the context of the specific assay employed.

The POC test, used in the current study, provides immediate results, enabling rapid clinical decision-making without the need for external laboratory analysis, making it an easy technique for clinicians in the field. Validation studies demonstrate a fair correlation between this POC test and reference assays such as turbidimetric immunoassay (r_s_ = 0.69), but direct comparison between both techniques is not recommended.^28^^,^^29^ Furthermore, discrepancies might arise, with the POC test, at high SAA concentrations due to the hook effect.^28^ Additionally, the matrix used for analysis (whole blood vs. serum) can influence measured values.^30^ In this study, whole blood was consistently used to reflect field conditions, as this approach is most practical for first-line practitioners.

In contrast to the admission measurement, on the second day of hospitalization only neutropenia positivity and combination of neutropenia, blood culture positivity, or both was associated with higher SAA concentrations. In this cohort, analysis yielded an optimal cut-off value of 933 μg/mL on day 2 for combined neutropenia, blood culture positivity, or both. This cut-off showed only a moderate diagnostic performance for sepsis. This could reflect the limited sensitivity and specificity of blood cultures, potentially misclassifying truly septic foals, or indicate that neutrophil changes occur earlier, with a delayed SAA response. To the authors knowledge no studies have yet compared the temporal dynamics of neutrophils and SAA during inflammation or infection in horses.

Brier scores ranged from 0.15 to 0.23 on day 0 and from 0.20 to 0.25 on day 2, consistent with modest overall predictive accuracy. Sepsis defined as the presence of neutropenia and the combination of neutropenia, blood culture positivity, or both, yielded the most favorable concentration at baseline, whereas performance decreased slightly by day 2 for all definitions. These results align with the results of the logistic regression. The LR+ and LR-, as well as the Brier scores, reveal only a modest correlation of sepsis with SAA. These results indicate that the biomarker provides some diagnostic insight but does not function as a highly accurate standalone indicator.

In septic foals SAA concentrations remained elevated and stable during the first 48 hours, while the group of hospitalized foals showed rising SAA concentrations. As all foals were critically ill, broad-spectrum antimicrobials were immediately administered, pending bacteriological results. The stability of SAA in septic foals could suggest stabilization of the inflammatory process or bacterial sensitivity to the administered antimicrobials. In contrast, the increase in SAA in the hospitalized group might reflect sampling in early phase of an inflammatory response or development of health-care associated infections (HAIs), which could cause an increase in SAA. It is assumed that, similar to findings in human medicine, hospitalized foals are at risk to develop HAIs.^31^ Additionally, it is possible that, as observed in human medicine, inflammatory responses can vary depending on the causative agent, leading to different concentrations of certain biomarkers.^32^ Additionally, all foals underwent catheterization and multiple venipunctures, which both can induce inflammation and potentially raise SAA concentration, to the authors knowledge, data on these possible effects are currently lacking. Finally, false-negative bacteriological results during the early phase of sepsis cannot be excluded in the hospitalized group. Another possible explanation for the increase in SAA concentration in the hospitalized group might be, at least in part, a physiological rise in SAA typically observed in healthy foals during the first 72 h of life, with peak concentrations occurring around 48 hours after birth.^17^^,^^25^^,^^33^ While serial SAA measurements are commonly used in adult horses to monitor treatment response our results suggest that in neonatal foals serial SAA testing during the first 48 hours might not provide useful additional diagnostic value for sepsis detection or prognosis.^22^^,^^34^

Regarding risk of death, SAA concentration did not appear related to outcome, either upon admission or at day 2 or repeatedly measured. When sensitivity analysis was performed excluding all euthanized foals the effect on outcome approached statistical significance (*P* = .05), but HR was extremely close to 1, suggesting a limited clinical relevance. These results are in contrast to the findings of a recent study on a larger group of foals in the same institute where SAA values >2054 μg/mL at hospital admission were associated with an increased risk of death.^35^ The larger sample size at the same institution, as well as the younger age (less than 1 week of age) of the foals in the previous study might explain these different observations. This highlights the need for additional biomarkers to improve prognostic assessment in critically ill neonatal foals. However, in our study there was no differentiation between foals that were euthanized for medical, prognostic, or financial reasons, which could have influenced the outcome analysis.

The present study was constrained by a number of limitations. First, the definition of sepsis remains challenging, even in human medicine. Recently a working group has been established with the aim of developing consensus definitions for foal sepsis, as well as coordinating the development of new scoring systems for sepsis and risk of death.^26^ Interpretation of SAA values across studies is further complicated by the lack of consensus on the definition of neonatal sepsis. Differences in inclusion criteria and diagnostic standards hinder comparisons and limit the establishment of universal reference intervals.^5^^,^^36–38^ The development of consensus definitions marks progress, facilitating cross-study comparisons, which is currently challenging. In the present study, sepsis was defined by being critically ill combined with a positive blood culture. However, blood culture has limited sensitivity and specificity, particularly in neonates.^7^ Single-timepoint blood sampling and culture has a moderate to low sensitivity and specificity, and prior antimicrobial treatment, the timing of sampling or small blood collection volumes reduce the likelihood of bacterial isolation.^7^ To mitigate this effect, 2 venipunctures and 3 culture vials (2 aerobic, 1 anaerobic) were used to maximize blood volume and culture yield.^7^ Despite these efforts, some septic foals might have been misclassified as hospitalized. Therefore additional definitions of sepsis have been added in the analysis. According to the latest working group, a sepsis definition has proposed, including 2 components: a life threatening condition involving proven or suspected infection, combined with clinical manifestations of infection. Proven infection is defined as a positive blood culture or positive culture from 2 different sites, whereas suspected infection was described as the presence of neutropenia and left shift. Systemic manifestations are yet to be clearly determined. Based on these criteria, the current study included presence of neutropenia and SIRS to help account for potential misclassification of septic foals solely on blood culture. Toxic changes to neutrophils were not included in the diagnostic criteria for the current study, as blood smears were not always performed on the moment of admission and quality of cell morphology was inconsistent. Furthermore, when interpreting a positive blood culture, distinguishment of pathogenic isolates from contaminants remains a challenge, especially since transient bacteremia can occur in clinically healthy neonatal foals.^5^^,^^7^^,^^39^ Another limitation was the missing data on day 2 of hospitalization, which was mainly caused by death before assessment. Death was closely associated with the severity of clinical disease, therefore it represents a missing non-ad random (MNAR) pattern. Standard imputation methods rely on missing ad random (MAR) patterns and can therefore introduce bias when MNAR mechanisms are applied. For this reason, the study used survival-sensitivity analysis. In addition the relative small sample size on day 2, might limit the precision of some estimates, which means results should be interpreted with caution.

The moderate to high sensitivity of the admission SAA cut-off supports its use as a complementary screening tool in neonatal foals at risk of sepsis, particularly when used alongside clinical assessment. However, clinicians should be cautious in interpreting elevated SAA concentrations without integrating additional clinical findings.^22^ Physiological increases in SAA occur in healthy neonatal foals, typically peaking around 48 h of age.^17^^,^^25^^,^^33^ This highlights the need to account for age when interpreting inflammatory markers, a strategy already adopted for lactate and leukocyte count in neonatal SIRS criteria.^8^

In summary, sequential measurements of SAA within the first 48 h did not provide additional diagnostic or prognostic value in this single-institution cohort. While SAA might assist in early screening for neonatal sepsis, it should not be used as a single test to guide antimicrobial therapy given the large number of false positives and false negatives. Serum amyloid A measurement at hospital admission with a cut-off value <419 μg/mL, performed using a POC test, showed moderate diagnostic performance as a single biomarker in the present cohort of critically ill neonatal foals. Its clinical utility to definitively rule in or rule out sepsis was limited, indicating that a single dichotomous SAA measurement is insufficient for clinical decision-making. This information might be useful for the ongoing initiatives on consensus definitions of sepsis and development of sepsis and survival scores.

## Supplementary Material

appendix_aalag155

Protocol_Eng_aalag155

## Off-label antimicrobial declaration

Authors declare no off-label use of antimicrobials.

## Institutional animal care and use committee or other approval declaration

Approved by the Ethical Committee of the Faculty of Veterinary Medicine and Bioscience Engineering, Ghent University (EC2021/023).

## Abbreviations

AUC: area under the curve
CI: confidence interval
HR: hazard ratio
IQR: interquartile range
LR+: positive likelihood ratio
LR-: negative likelihood ratio
MALDITOF-MS: matrix-assisted laser desorption/ionization time of flight spectrometry
NMS: Neonatal maladjustment syndrome
NPV: negative predictive value
OR: odds ratio
POC-test: point-of-care test
PPV: positive predictive value
ROC: receiving operating characteristics
Se: sensitivity
SE: standard error
Sp: specificity
