# Supplementary material for: Evaluation of sequential serum amyloid A measurements for the prediction of sepsis and survival in critically ill neonatal foals
Source: J Vet Intern Med. 2026 Aug 6;40(4):aalag155. doi: 10.1093/jvimsj/aalag155 (PMC13446502; doi:10.1093/jvimsj/aalag155)
Supplement: Supplementary file 1 [file appendix_aalag155.docx]

**Appendix**

*Study design, sample size and cohort*

A convenience sample was used and the inclusion criteria consisted of critically ill neonatal foals less than 14 days old. Critical illness was defined as the presence of clinical signs compatible with neurological dysfunction (lethargy or coma) with or without a decreased or absent suckle reflex and the need for intravenous fluid support. The need for intravenous fluid therapy was based on clinical parameters at the discretion of the treating clinician: increased heart rate, pulse quality, cold extremities, mucous membranes, abnormal mentation or decreased blood pressure (indirect measurement), or both, combined with blood parameters (increased lactate concentration, electrolyte and acid-base imbalances. Within this critically ill study cohort, foals were defined as septic within the present study, when blood culture vials tested positive. Because blood culture alone might underestimate presence of sepsis, additional analysis were performed using alternative sepsis definitions: blood culture positivity, neutropenia, using age-specific ranges, and systemic inflammatory response syndrome (SIRS). Foals were classified as SIRS positive when at least two of the following six criteria were present: rectal temperature (<37.2 °C or >39.2 °C), tachycardia (>115 beats per minute (bpm) in newborn foals and >120 bpm in neonatal foals), tachypnoea (>56 breaths per minute), abnormal leukocyte count, increased venous L-lactate concentration (>5 mmol/L in newborn foals and >2.5 mmol/L in neonatal foals), or abnormal venous blood glucose concentration (<50 or >160 mg/dL in newborn foals and <75 or >160 mg/dL in neonatal foals). Neutropenia was defined using age-specific reference ranges for circulating neutrophil counts: <4.1 ×10⁹/L for foals at 1 day of age, <5.0 ×10⁹/L for 2 days of age, and <6.6 ×10⁹/L for foals aged 2–14 days.^41^ Two composite groups were constructed: one including foals fulfilling at least one criterion for blood culture positivity or neutropenia, and another including foals fulfilling at least one criterion for blood culture positivity or SIRS.

Data were collected by nine experienced equine clinicians. A standardized evaluation form was used for data collection. A detailed history was collected, including information on periparturient details (gestational length, illness before parturition, premature lactation, presence of placentitis, dystocia, red bag delivery, and non-survival of the mare), as well as medical treatments provided before referral (such as antimicrobials, anti-inflammatory drugs, plasma, etc.). The signalment (breed, sex and age) of the foal was recorded. Twelve clinical variables were recorded, including cardiac parameters (heart rate and rhythm, presence or absence of a murmur), respiratory rate, lung auscultation (normal, wheezes or crackles), rectal temperature (°C), mental state, color of mucous membranes, posture, presence of cold extremities, presence or absence of a suckle reflex, presence of localized infections (enteritis, pneumonia, umbilical infection, septic arthritis and presence of wounds). Based on clinical findings, foals were diagnosed with prematurity, dysmaturity, neonatal maladjustment syndrome (NMS), general weakness, sepsis, enteritis, pneumonia, and other non-infectious pathologies (including hemorrhage, myopathy and trauma).

On admission, the jugular vein was clipped, scrubbed with a 4% chlorhexidine solution and disinfected with a 70% isopropanol solution. Blood for culture was aseptically sampled with a 21G needle before placing the intravenous catheter. A total of 30 mL of blood was collected by two venipunctures and aseptically divided over two aerobic and one anaerobic 8-10 mL vials (BD-BACTEC^TM^). SAA concentration was determined on EDTA-anticoagulated whole blood and measured by using a point-of-care (POC) test (Stablelab EQ-1 handheld reader, Zoetis B.V., Parsippany, New Jersey, United States) within 30 minutes after blood collection. The blood culture vials were incubated in an automated system at 35°C for detection of microbial growth (BACTEC^TM^ FX, BD, Erembodegem, Belgium). Negative cultures were defined by the absence of bacterial growth after five days of incubation and no colony formation on Columbia blood, Columbia nalidixic acid and chocolate agars after 48 hours. Positive samples were further characterized by matrix-assisted laser desorption/ionization time of flight mass spectrometry (MALDITOF-MS).

On day 2 of hospitalization whole blood was sampled with a 21G needle from the jugular, cephalic or saphenous vein and collected in an EDTA-tube to determine SAA concentration as described above. Clinical outcome was defined as survival to discharge from the hospital or mortality. Regarding mortality, a distinction was made between natural death and euthanasia. No distinction was made between foals that were euthanized for medical, prognostic or financial reasons.

*Statistical analysis*

Data were collected in a worksheet (Excel, Microsoft Inc, Washington) and transferred to a statistical program (IBM SPSS Statistics for Windows, Version 29.0. Armonk, NY: IBM Corp) for analysis. SAA values were tested for normality using visual inspection of histograms, Q-Q plots and the Shapiro-Wilk test. Data were non-normally distributed and are presented as median and interquartile range [Q1; Q3]. The association of SAA concentration at the two time points with sepsis was evaluated using logistic regression. In a first analysis, the association of SAA with sepsis, using four different definitions, was determined for each time point (arrival and after 48 hours) separately. Receiver operating characteristic (ROC) curve analysis and the Youden index were used to determine optimal cut-off values for SAA concentration, when significant. Model fit was assessed using the Hosmer-Lemeshow test. In a second analysis, the difference in SAA concentration between arrival and day two of hospitalization was calculated. The difference in SAA concentration was assessed as a binary variable in a logistic regression model. A threshold of 50 µg/mL was applied to determine meaningful change. SAA concentrations differing by ≥50 µg/mL were categorized as positive or negative changes, whereas differences <50 µg/mL were considered as no change. Multivariable logistic regression was performed to evaluate the association of SAA (arrival, day 2 and the difference in SAA) as a binary variable with the sepsis under the different definitions. Models were adjusted for potential confounders, including, age, prematurity, prior antimicrobial treatment and presence of comorbidities. In a third analysis, a logistic regression model with sepsis as binary outcome was used to compare previously determined cut-off values from previous studies on the current cohort.^17,18^

To study the association of SAA with death, univariable Cox proportional hazard (survival) analysis was performed. The event of interest was death (natural or euthanasia), and SAA was considered as predictor. Time-to-event was recorded from arrival to either death or discharge. Right censoring was applied the day of hospital discharge. In a first approach, a separate analysis for SAA concentration was performed on hospital arrival and 48 hours of hospitalization. In a second analysis, a sensitivity analysis was conducted in which euthanized foals were excluded as non-survivors to evaluate the impact of euthanasia on survival. In a third analysis, the difference between arrival concentrations and after 48 hours concentrations were assessed as a binary variable in a logistic regression model. A threshold of 50 µg/mL was applied to determine meaningful change. SAA concentrations differing by ≥50 µg/mL were categorized as positive or negative changes, whereas differences <50 µg/mL were considered as no change. The proportional hazard assumption was visually assessed using log-minus-log plots and by incorporating time-varying covariates into the model. The overall significance level was set at *p* <.05.

Diagnostic performance of SAA as predictor of sepsis or mortality was assessed by calculating sensitivity (Se), specificity (Sp) and accuracy). In addition, the positive likelihood ratio (LR+) defined as $\frac{sensitivity}{1-specificity}$, and the negative likelihood ratio (LR-) defined as $\frac{1-sensitivity}{specificity}$ were calculated.^42^ Furthermore, the predictive accuracy of SAA was assessed using the Brier score. To quantify uncertainty, 2000-sample non-parametric bootstrapping was performed to obtain bias-corrected confidence intervals.

**References**

1. Cohen ND. Causes of and farm management factors associated with disease and death in foals. J Am Vet Med Assoc 1994;204:1644-1651.

2. Eaton S. Neonatal sepsis – Pathology and clinical signs. Equine Veterinary Education 2023;35:498-503.

3. Taylor S. A review of equine sepsis. Equine Veterinary Education 2015;27:99-109.

4. Wong D, Wilkins PA, Landolt G. Neonatal Infection. In: Equine Neonatal Medicine2024:1126-1177.

5. Corley KTT, Furr MO. Evaluation of a score designed to predict sepsis in foals. Journal of Veterinary Emergency and Critical Care 2003;13:149-155.

6. Palmer J. Update on the management of neonatal sepsis in horses. Vet Clin North Am Equine Pract 2014;30:317-336, vii.

7. Giancola S, Hart KA. Equine blood cultures: Can we do better? Equine Veterinary Journal 2023;55:584-592.

8. Celik IH, Hanna M, Canpolat FE, et al. Diagnosis of neonatal sepsis: the past, present and future. Pediatr Res 2022;91:337-350.

9. Eichberger J, Resch E, Resch B. Diagnosis of Neonatal Sepsis: The Role of Inflammatory Markers. Front Pediatr 2022;10:840288.

10. Yuan H, Huang J, Lv B, et al. Diagnosis value of the serum amyloid A test in neonatal sepsis: a meta-analysis. Biomed Res Int 2013;2013:520294.

11. Gutiérrez-Pizarraya A, León-García MDC, De Juan-Idígoras R, et al. Clinical impact of procalcitonin-based algorithms for duration of antibiotic treatment in critically ill adult patients with sepsis: a meta-analysis of randomized clinical trials. Expert Rev Anti Infect Ther 2022;20:103-112.

12. Schuetz P. How to best use procalcitonin to diagnose infections and manage antibiotic treatment. Clin Chem Lab Med 2023;61:822-828.

13. Schuetz P, Birkhahn R, Sherwin R, et al. Serial Procalcitonin Predicts Mortality in Severe Sepsis Patients: Results From the Multicenter Procalcitonin MOnitoring SEpsis (MOSES) Study. Crit Care Med 2017;45:781-789.

14. Bonelli F, Meucci V, Divers T, et al. Evaluation of Plasma Procalcitonin Concentrations in Healthy Foals and Foals Affected by Septic Systemic Inflammatory Response Syndrome. Journal of Equine Veterinary Science 2015;35:645-649.

15. Pusterla N, Magdesian KG, Mapes S, et al. Expression of molecular markers in blood of neonatal foals with sepsis. American Journal of Veterinary Research 2006;67:1045-1049.

16. Zabrecky KA, Slovis NM, Constable PD, et al. Plasma C-Reactive Protein and Haptoglobin Concentrations in Critically Ill Neonatal Foals. Journal of Veterinary Internal Medicine 2015;29:673-677.

17. Barr B, Nieman NM. Serum amyloid A as an aid in diagnosing sepsis in equine neonates. Equine Vet J 2022;54:922-926.

18. Hoeberg E, Sånge A, Saegerman C, et al. Serum amyloid A as a marker to detect sepsis and predict outcome in hospitalized neonatal foals. J Vet Intern Med 2022;36:2245-2253.

19. Hurcombe SD, Toribio RE, Slovis NM, et al. Calcium regulating hormones and serum calcium and magnesium concentrations in septic and critically ill foals and their association with survival. J Vet Intern Med 2009;23:335-343.

20. Rieger M, Kochleus C, Teschner D, et al. A new ELISA for the quantification of equine procalcitonin in plasma as potential inflammation biomarker in horses. Anal Bioanal Chem 2014;406:5507-5512.

21. Belgrave RL, Dickey MM, Arheart KL, et al. Assessment of serum amyloid A testing of horses and its clinical application in a specialized equine practice. J Am Vet Med Assoc 2013;243:113-119.

22. Jacobsen S. Use of serum amyloid A in equine medicine and surgery. Veterinary Clinical Pathology 2023;52:8-18.

23. Witkowska-Piłaszewicz OD, Żmigrodzka M, Winnicka A, et al. Serum amyloid A in equine health and disease. Equine Vet J 2019;51:293-298.

24. Yu MH, Chen MH, Han F, et al. Prognostic value of the biomarkers serum amyloid A and nitric oxide in patients with sepsis. Int Immunopharmacol 2018;62:287-292.

25. Witkowska-Piłaszewicz OD, Żmigrodzka M, Winnicka A, et al. Serum amyloid A in equine health and disease. Equine Veterinary Journal 2019;51:293-298.

26. Stoneham SJ, Palmer L, Cash R, et al. Measurement of serum amyloid A in the neonatal foal using a latex agglutination immunoturbidimetric assay: determination of the normal range, variation with age and response to disease. Equine Vet J 2001;33:599-603.

27. Wilkins PA, Wong DM. Report from the 2024 Dorothy Russell Havemeyer Working Group Meeting on Consensus Definitions for Foal Sepsis. Equine Vet J 2025;57:536-539.

28. van den Brom-Spierenburg AJ, Siegers EW, Westermann CM, et al. Sequential measurement of serum amyloid A concentrations in ill hospitalized neonatal foals. Journal of Veterinary Internal Medicine 2026;40.

29. Kiemle J, Hindenberg S, Bauer N, et al. Comparison of a point-of-care serum amyloid A analyzer frequently used in equine practice with 2 turbidimetric immunoassays used in human and veterinary medicine. J Vet Diagn Invest 2022;34:42-53.

30. Schwartz D, Pusterla N, Jacobsen S, et al. Analytical validation of a new point-of-care assay for serum amyloid A in horses. Equine Vet J 2018;50:678-683.

31. Slovis NM, Browne N, Bozorgmanesh R. Point-of-Care Diagnostics in Equine Practice. Vet Clin North Am Equine Pract 2020;36:161-171.

32. Theelen MJP, Wilson WD, Byrne BA, et al. Differences in isolation rate and antimicrobial susceptibility of bacteria isolated from foals with sepsis at admission and after ≥48 hours of hospitalization. J Vet Intern Med 2020;34:955-963.

33. Leli C, Ferranti M, Moretti A, et al. Procalcitonin Levels in Gram-Positive, Gram-Negative, and Fungal Bloodstream Infections. Disease Markers 2015;2015:701480.

34. Palmisano M, Javsicas L, McNaughten J, et al. Effect of plasma transfusion on serum amyloid A concentration in healthy neonatal foals and foals with failure of transfer of passive immunity. J Vet Intern Med 2023;37:697-702.

35. Long A, Nolen-Walston R. Equine Inflammatory Markers in the Twenty-First Century: A Focus on Serum Amyloid A. Veterinary Clinics: Equine Practice 2020;36:147-160.

36. Castelain DL, Dufourni A, Pas ML, et al. Retrospective cohort study on diseases and risk factors associated with death in hospitalized neonatal foals. Journal of Veterinary Internal Medicine 2025;39:e17269.

37. Dunkel B, Corley KTT. Pathophysiology, diagnosis and treatment of neonatal sepsis. Equine Veterinary Education 2015;27:92-98.

38. Hayes R, Hartnett J, Semova G, et al. Neonatal sepsis definitions from randomised clinical trials. Pediatr Res 2023;93:1141-1148.

39. Sheats MK. A Comparative Review of Equine SIRS, Sepsis, and Neutrophils. Front Vet Sci 2019;6:69.

40. Furr M, McKenzie H, 3rd. Factors associated with the risk of positive blood culture in neonatal foals presented to a referral center (2000-2014). J Vet Intern Med 2020;34:2738-2750.

41. Felippe MJB. Evaluation of the Hematopoietic System – Flow Cytometry. In: Equine Neonatal Medicine2024:1055-1059.

42. Grimes DA, Schulz KF. Refining clinical diagnosis with likelihood ratios. Lancet 2005;365:1500-1505.
