# Supplementary material for: Evaluation of sequential serum amyloid A measurements for the prediction of sepsis and survival in critically ill neonatal foals
Source: J Vet Intern Med. 2026 Aug 6;40(4):aalag155. doi: 10.1093/jvimsj/aalag155 (PMC13446502; doi:10.1093/jvimsj/aalag155)
Supplement: Supplementary file 2 [file protocol_eng_aalag155.docx]

Identification patiënt

**Form: critically ill foals**

|  | Critically ill / Sick / Healthy |
| --- | --- |
| **Date (blood culture)** | ......................................................................................  2x Aerobic  1x Anaerobic |
| **Prior treatment** | Y / N  If yes, what medication has been given:   - NSAIDs - Antimicrobials: - Corticosteroides - Plasma - Others.. |
| **Sample at arrival?** | Y / N, after …. Days of hospitalisation |
| **Prematurity** | Premature / a term / Prolonged gestation   - 1 week - 2 weeks - 3 weeks - > 3 weeks |
| **Clinical examination foal** |  |
| Posture and decubitus | Standing – Sternal - lateral  Standing after stimulation? Y / N  Alert – hypotonia – lethargy – coma – seizures – floppy ears |
| Mucous membranes | - Pale - Light pink - Pink - Congested - Icteric - Petechia - Cyanotic   CRT: |
| Suckle reflex | Absent – weak – normal |
| Eyes | - *Scleral vessel injections: Present / Absent - *Anterior uveitis: Present / Absent - Entropion: Present / Absent - Nystagmus: Present / Absent - Palpebraalreflex: Present / Absent   * not due to trauma or other underlying eye disease |
| Respiration | Dyspnea : Present / Absent  Lung auscultation: Normal / Abnormal  If abnormal, describe |
| Cardiovascular | Heart auscultation: Normal / Abnormal  If abnormal, describe  Pulse: Hyperkinetic / normal / zwak / weak / other (describe)  MAP (mean arterial pressure):......................mmHg |
| Temperature | °C |
| Extremities | Cold / Normal |
| Musculoskeletal | - Tendon retraction : Present / Absent - Tendon elongation: Present / Absent - Hoofs matuer: Y / N - Poor perfusion coronet: Present / Absent |
| Eye position | Deep / Normal |
| Focal infection | Present / Absent  If present, describe:   - Distended joint:   - 1 joint   - 2 or more joints - Umbilicus   - Normal (dry and not swollen)   - Swollen but dry   - Swollen and wet (blood, pus or urine)   - Describe ultrasound: - Diarrhea - Pneumonia (describe ultrasound) |
| Meconium | Passed / Not passed |
| Urinary | Urinedensity:.............................................................................Colour urine:..............................................................................  Polyuria – anuria – oliguria – hematuria – other:…............................................................................. |
| Wounds | Present / Absent |
| Post-partum events | Standing up after ……………… h  Urination after ………………..h  Suckling after ………………….h |
| **Examination mare** |  |
| Peripartum events mare | - Placentitis - Dystocia - Disease in last month of gestation - Vaginal discharge prepartum - Induction partus - Caesarian - Long transport prepartum (> 3 month) - Prepartum lactation - Prematurity   Number previous foals ………………….. |
| Placenta passed ? | Y / N |

**Comments**
